# Neurostimulation in the treatment of primary headaches

**DOI:** 10.1136/practneurol-2015-001298

**Published:** 2016-05-05

**Authors:** Sarah Miller, Alex J Sinclair, Brendan Davies, Manjit Matharu

**Affiliations:** 1 Headache Group, Institute of Neurology and The National Hospital for Neurology and Neurosurgery, London, UK; 2 Neurometabolism, Institute of Metabolism and Systems Research, College of Medical and Dental Sciences, The University of Birmingham, Birmingham, UK; 3 Department of Neurology, Royal Stoke University Hospital, Stoke-on-Trent, UK

**Keywords:** HEADACHE, MIGRAINE, Neurostimulation

## Abstract

There is increasing interest in using neurostimulation to treat headache disorders. There are now several non-invasive and invasive stimulation devices available with some open-label series and small controlled trial studies that support their use. Non-invasive stimulation options include supraorbital stimulation (Cefaly), vagus nerve stimulation (gammaCore) and single-pulse transcranial magnetic stimulation (SpringTMS). Invasive procedures include occipital nerve stimulation, sphenopalatine ganglion stimulation and ventral tegmental area deep brain stimulation. These stimulation devices may find a place in the treatment pathway of headache disorders. Here, we explore the basic principles of neurostimulation for headache and overview the available methods of neurostimulation.

## Background

Headache conditions can be classified into episodic and chronic forms (box 1). Chronic headache is a major global health issue affecting up to 4% of the population.^1^ Most patients with chronic headache attending neurology centres have chronic migraine or cluster headache. The estimated prevalence of chronic migraine is 2% and of chronic cluster is 0.02%.^2^ Although most patients are helped with medical treatments, a significant minority cannot tolerate or prove refractory to pharmacological treatments. Neurostimulation therapies with peripheral or central targets may help these patients. Here, we provide an overview of this emerging field of headache management.

Box 1 **Classification of episodic and chronic headache**

Episodic headache

Headache occurs on <15 days a month for >3 months.

Chronic headache

Headache occurs on >15 days a month for >3 months.

## Neurostimulation for pain

Electrical stimulation to treat pain is not a new concept. Scribonius Largus, ca. CE 46, advocated fish ‘electrotherapy’ to treat headache.^3^ Modern neurostimulation for pain has its roots in functional neurosurgery, which initially relied on destructive lesions before focusing on neurostimulation to manage intractable pain. Neurostimulation works by manipulating central or peripheral pain pathways using electrical or magnetic impulses. It aims is modify the pain system in such a way to reduce pain levels.

**Figure 1:**
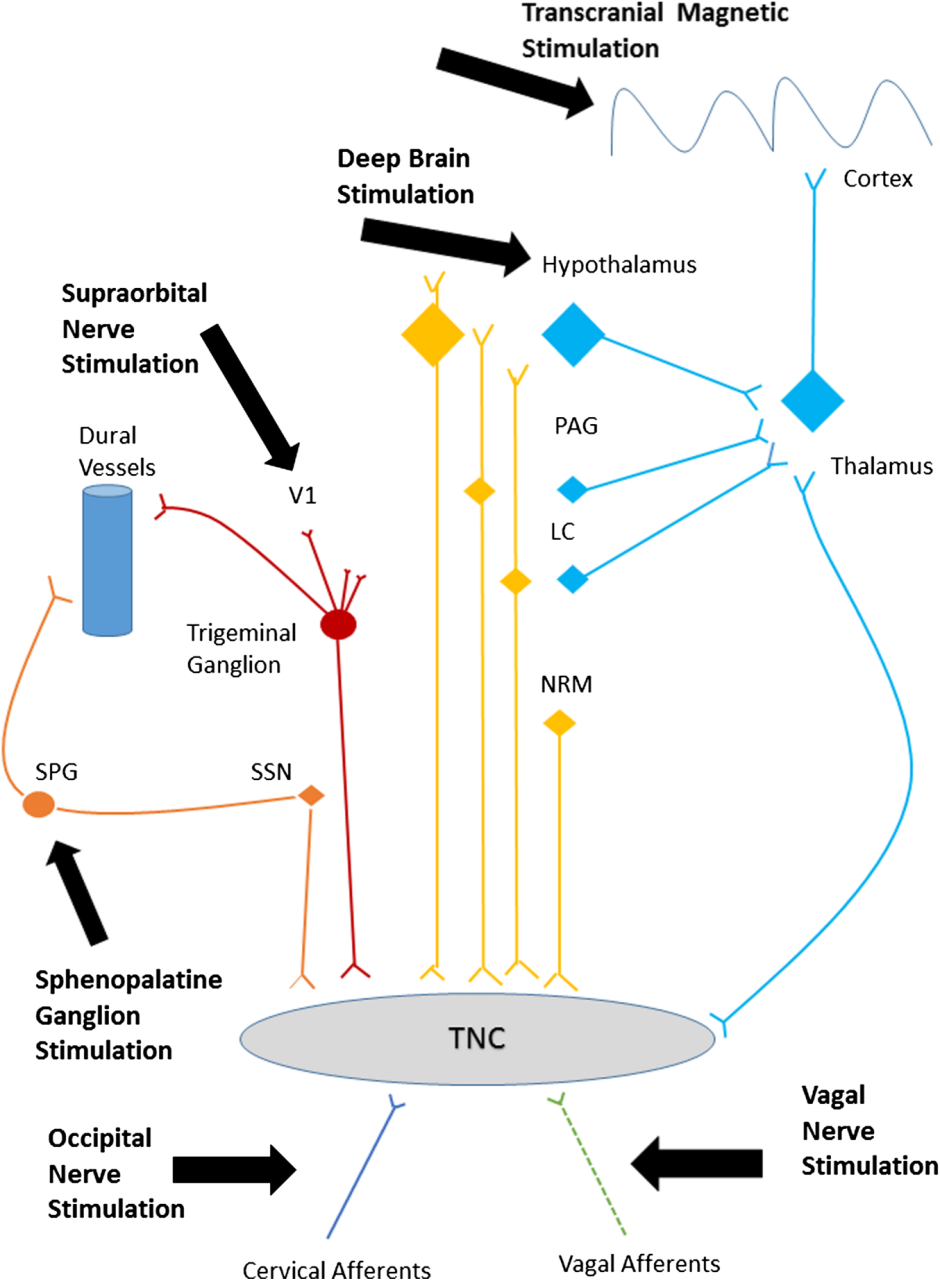
Headache pain pathways targeted by neurostimulation. A simplified diagram of the various peripheral and central pain pathways targeted by current neurostimulation devices. LC, locus coeruleus; NRM, nucleus raphe magnus; PAG, periaqueductal grey; SPG, sphenopalatine ganglion; SSN, superior salivary nucleus; TNC, trigeminal nucleus caudalis; V1, first division of the trigeminal nerve.

There are multiple targets for neurostimulation in headache, including the posterior hypothalamus/ventral tegmental area, sphenopalatine ganglion (SPG), occipital nerve, vagus nerve, supraorbital nerve and cortex. These have been selected because of the recent recognition of anatomically relevant pathophysiological pathways and hypothesised mechanisms in both animal and human models of migraine and cluster headache. Applying electrical or magnetic stimulation to pain pathways can modify central neurotransmitters.^4^ For preventive treatment, these changes aim to cause a wind down of the central sensitisation that occurs in chronic headache. For acute treatment, these changes probably block the processes responsible for attack generation (cortical spreading depression or brainstem activation). Figure 1 summarises the pathways most likely involved in the mechanism of action of these neurostimulation treatments.

Currently available non-invasive devices target the supraorbital and vagus nerves (electrical stimulation) or the cortex (magnetic stimulation). Available invasive stimulation techniques involve the occipital nerves and SPG (peripheral targets) and the ventral tegmental area (central target). Such techniques may help those patients who wish to avoid, are refractory to or intolerant of previous drug therapies. Devices that allow acute treatment of attacks may be help those who cannot use triptans or in whom acute medications are ineffective or overused. Pregnancy—another situation where it may be difficult to use preventative and acute headache treatments—may, in theory, be a specialised situation where neurostimulation devices could be used. However, the safety of such devices in pregnancy is not established. Laboratory data suggest that exposure to electrical stimulation techniques is safe in animal studies and limited open-label studies suggest safety in occipital nerve stimulation and transcranial magnetic stimulation.

## Non-invasive neurostimulation devices

### Supraorbital nerve stimulation and the Cefaly device

**Figure 2:**
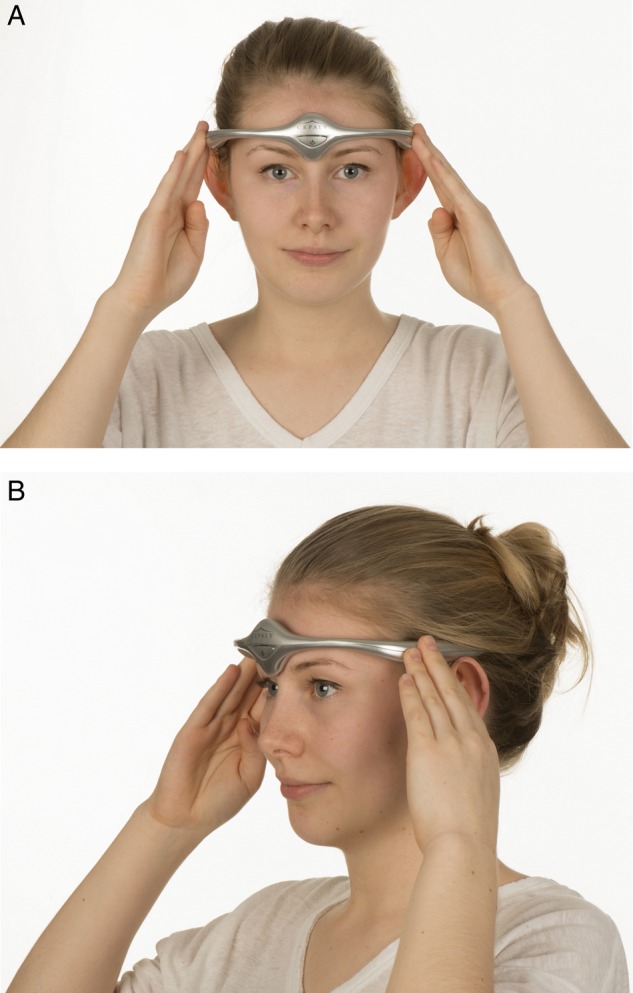
Non-invasive supraorbital nerve stimulation. (A) and (B) show how a patient would use the Cefaly device to deliver preventative treatment for migraine. Image published with permission of individual.

The trigeminovascular system has a crucial role in head pain.^5^ The supraorbital nerve is a branch of the first division of the trigeminal nerve. Transcutaneous supraorbital nerve stimulation has been developed as a potential treatment for headache. The Cefaly device is an external transcutaneous supraorbital nerve stimulator that is battery powered and applied to the forehead using a headband-like device (figure 2). Currently, the device is not available on the National Health Service (NHS) but can be purchased directly from the manufacturer. The cost is £249 for the device and around £50 for a 3-month supply of reusable electrodes.

#### Possible role for the Cefaly device

##### Acute treatment of episodic migraine

There is no well-controlled evidence for using the Cefaly device in the acute treatment of episodic migraine. A single pilot study of 10 patients using the device for acute migraine treatment showed no effect on 57% of treated attacks and was associated with pain freedom in only 13% of attacks.^6^

##### Preventative treatment of episodic migraine

Evidence for the use of the Cefaly device in preventing episodic migraine comes from one small, manufacturer-sponsored sham-controlled trial, the PREvention of MIgraine using the STS Cefaly (the PREMICE study) and company postmarketing survey data. The sham-controlled study involved only 67 subjects with episodic migraine, who after a one month run-in period of normal treatment used the Cefaly or sham device for 3 months and reported a significant reduction in migraine days by 29.7% from 6.94 to 4.88 days (p=0.023) in the active group compared to a non-significant change of 4.9% from 6.54 to 6.22 days (p=0.608) in the sham group.^7^ For comparison, topiramate 100 mg daily decreased migraine days by 44% in a pooled analysis of controlled studies.^8^ The therapeutic gain for the 50% responder rate of supraorbital nerve stimulation was 26.1% compared with 23.5% for topiramate but the therapeutic gain in migraine day reduction is much higher in pooled studies of topiramate at 24.5% compared with the PREMICE trial at 12%.^8^ The responder rate shown in the PREMICE study was within the range quoted for other migraine preventatives such as propranolol and antiepileptic medications.^9^ ^10^ The postmarketing survey of 2313 subjects using the device to prevent episodic migraine reported 53% of users were ‘satisfied’ with the treatment, as determined by the number continuing treatment after a 40-day trial period.^11^ In the PREMICE trial, 70.6% declared themselves satisfied after 3 months of treatment.^7^ In contrast, using stopping of treatment as a measure of patient satisfaction, one American health insurance survey of migraine preventatives found that 26.6% of patients on antidepressants, 29.8% on antiepileptic medications and 32.4% on beta-blockers were satisfied and remained on treatment after 6 months.

#### Using the Cefaly device

Box 2 outlines a preventative regimen involving preset stimulation sessions to be used daily. As with all migraine preventatives, treatment must continue for 3 months before assessing efficacy, so encouraging treatment adherence is important. The manufacturers have a preprogrammed 20 min session ‘acute setting’ for terminating a migraine attack, despite there being no evidence to support its use in acute migraine.

Box 2 **Using the Cefaly device to prevent episodic migraine**

Place sticky electrodes on the forehead and attach the device to the electrode (figure 2).

For preventative treatment of episodic migraine:

To deliver the preset preventative mode, press the device on-switch twice to deliver a 20 min session.

Patients should receive daily preventative sessions for at least 3 months before assessing efficacy.

The adverse effects of the Cefaly device appear mild and transient.^11^ Some patients experience intolerance to paraesthesia, drowsiness, worsening of headache and reversible forehead irritation. Table 2 lists its contraindications.

#### Proposed mechanism of action

We do not yet know the mode of action of supraorbital nerve stimulation in migraine. In healthy volunteers, stimulation with supraorbital nerve stimulation has an acute sedative effect.^12^ Although it is questionable whether this influences the preventive treatment of migraine, it does suggest that supraorbital nerve stimulation can change brain activity. Current understanding of migraine pathophysiology suggests that there is neuronal hyperexcitability of the pain neuromatrix: trigeminovascular activation appears to be the basis for the headache phase and central sensitisation the mechanism that may contribute to the transformation from episodic to chronic forms. Supraorbital nerve stimulation probably winds down the trigeminal pain pathways through altering activity within the trigeminovascular system both peripherally and centrally.

### Vagus nerve simulation and the gammaCore device

**Figure 3:**
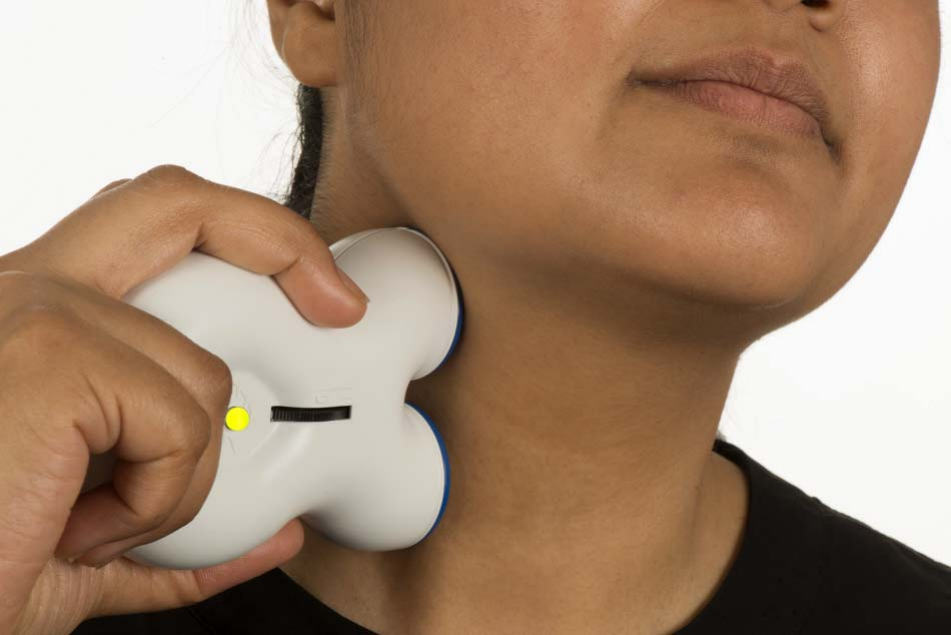
Non-invasive vagus nerve stimulation. How a patient would use the gammaCore device to deliver acute or preventative treatment for cluster headache. Image published with permission of individual.

The vagus nerve is a mixed motor and sensory nerve that is important in controlling autonomic responses; it projects to several higher centres that are important in pain regulation. Following reports of migraine improvement in patients receiving vagus nerve stimulation for epilepsy, the nerve became a target for headache treatment. The gammaCore device is a handheld transcutaneous vagus nerve stimulator applied to the neck (figure 3). Devices are available charged with either 150 or 300 treatment cycles, after which a new device must be purchased. If used for prevention only, the 300-treatment device lasts around 50 days; thus, a patient needs two devices to have the efficacy assessed at 3 months. Currently, the device is not available on the NHS but individual funding requests may be considered. If purchased direct from the manufacturer, the 300-treatment device costs around £550.

#### Evidence for the gammaCore device

##### Preventative treatment of cluster headache

The current evidence for its use in preventing cluster headache attacks is limited to a manufacturer-sponsored trial involving 97 subjects. This trial of standard of care plus vagus nerve stimulation versus standard of care alone was conducted on the preventative and acute treatment of chronic cluster headache using the gammaCore device. Regular use of gammaCore for 4 weeks was associated with a significantly reduced attack frequency in the active compared with standard of care group (7 vs 2 fewer attacks per week).^13^ Similarly, more subjects in the active group reported >50% fewer weekly attacks compared with those on standard treatment (52% vs 9%).^14^ For comparison, the responder rate for verapamil 360 mg daily was 80% versus 0% for placebo in the only available small randomised-controlled trial.^14^

##### Acute treatment of cluster headache

The above randomised study found no effect of acute gammaCore treatment on headache intensity.^13^ However, in a small open-label study of 19 patients using the device for acute treatment, 47% of attacks ended within 11 min (average duration before gammaCore use was 75 min).^15^ All 13 patients using triptans before starting using gammaCore treatment could reduce their intake, and four patients stopped triptans completely. The standard acute treatments for cluster headache attacks are subcutaneous sumatriptan and high-flow oxygen. In one randomised double-blind study of 39 patients, subcutaneous sumatriptan aborted 74% of attacks within 15 min compared with 26% of those treated with placebo.^16^ In another randomised control study of 76 patients, high-flow oxygen aborted 78% of attacks within 15 min, whereas placebo aborted only 20% of attacks.^17^

##### Preventative treatment of chronic migraine

There are no data to support the use of gammaCore to prevent chronic migraine. A single controlled trial of 59 patients comparing active gammaCore to sham treatment showed that, after 2 months, active treatment was not associated with a reduction of headache days (−1.9 days with gammaCore vs 0.20 sham (p=0.124)).^18^ Although open-label extension data suggest that a longer duration of treatment might be effective, we need more controlled studies to investigate this.

##### Acute treatment of migraine

There have been no controlled studies in acute migraine. Two small open-label studies examined gammaCore in treating acute migraine pain. The first, involving 27 patients treating 80 attacks, used two stimulation cycles administered 15 min apart during an attack.^19^ Twenty-two per cent of moderate-to-severe attacks achieved pain freedom within 2 h of treatment, a benefit similar to that of naproxen 500 mg. Another study of 24 patients with 79 attacks showed significantly reduced pain scores from baseline at 2 h following treatment, with a 50% reduction in 46% at 2 h, increasing to 61% at 4 h (report available only in poster form). In a randomised-controlled study of acute migraine treatment in 92 patients, 67% achieved a pain-free rate using 100 mg sumatriptan (placebo rate 28%).^20^ ^21^

#### Possible role of the gammaCore device

Current evidence suggests that the gammaCore device should only be considered to prevent cluster headache. There is no robust evidence for using it as an acute treatment (table 1). Nonetheless, it might be considered as an acute treatment for patients with more than two cluster attacks daily (ie, more attacks than can safely be treated with subcutaneous sumatriptan), in those with contraindications to triptans and in those where all acute medications for cluster headache are ineffective. There is no evidence as yet for using gammaCore in migraine.

**Table 1:** Neurostimulation and devices and the conditions in which they can be used

	Supraorbital stimulation	Vagus nerve stimulation	Transcranial magnetic stimulation	Occipital nerve stimulation	Sphenopalatine ganglion stimulation	Deep brain stimulation
Device name	Cefaly	gammaCore	SpringTMS		Pulsante	
Acute migraine attacks	X	✓	✓ (with and without aura)	X	X	X
Prevention of episodic migraine	✓	X	X	X	X	X
Prevention of chronic migraine	X	X	X	✓	X	X
Acute cluster attacks	X	✓	X	X	✓	X
Prevention of episodic cluster headache	X	✓*	X	X	X	X
Prevention of chronic cluster headache	X	✓	X	✓	✓ (studies ongoing)	✓
Other TACs	X	X	X	✓ (prevention of chronic intractable TACs)	X	✓ (prevention of chronic intractable TACs)

*Usefulness may be dictated by length of episodic cluster bout; if bout lasts <3 months, it may be difficult to assess as treatment may take this long to have a clear effect.

TAC, trigeminal autonomic cephalalgia.

#### Using the gammaCore device

Box 3 outlines our recommended regimens for using gammaCore. The activated device delivers a single programme cycle of 2 min. For preventative treatment of cluster headache, we recommend daily use of gammaCore for 3 months before assessing efficacy. If the patient wishes to use it for acute treatment of cluster headache, we recommend that they use it for two programme cycles at attack onset. The reported adverse effects are mild and include transient hoarseness, voice change, skin irritation, muscle ache and uncomfortable paraesthesia. Table 2 lists its contraindications.

Box 3 **Using the gammaCore device to treat cluster headache**

The patient locates their carotid pulse in the neck and places the device over this.

The device can be used on either side of the neck.

The stimulation is adjusted by turning a thumbwheel until a deep vibration is felt inside the neck. The patient should aim to use the maximum tolerable intensity (stimulation setting three or more in most people).

When correctly positioned, the subject should feel a pulling at the corner of the mouth. This is a normal response and patients should be encouraged to look out for it.

*For acute treatment of cluster headache attacks (evidence base poor)*:

Deliver two programme cycles (a total of 4 min) at the attack onset. Repeat after 15 min if needed.

*For preventative treatment of cluster headache attacks*:

Deliver one programme cycle (total of 2 min) three times a day.

The regimen can be increased to two cycles (total of 4 min) three times daily if required.

Preventative sessions should be delivered daily for 3 months before assessing efficacy.

**Table 2:** Contraindications for non-invasive neurostimulation

	Supraorbital stimulation	Vagus nerve stimulation	Transcranial magnetic stimulation
Device name	Cefaly	gammaCore	SpringTMS
Contraindicated for use	Recent brain or facial trauma (<3 months) Skin abrasion on the forehead in the area of application of the electrode Allergy to acrylate	Active implantable medical device, such as a pacemaker, defibrillator, cochlear implant and other implanted electronic device Metallic implants near the treatment site History of significant carotid atherosclerosis Cervical vagotomy	Active implantable medical device, such as a pacemaker, defibrillator, cochlear implant and other implanted electronic device Implants affected by a magnetic field†* Epilepsy
Cautious use	Electrohypersensitivity	Vasovagal syncope Skin irritation near the treatment site	Cardiac conditions

Devices should not be used while driving or operating machinery.

*Dental implant and fillings are not a contraindication to using transcranial magnetic stimulation.

†Including aneurysm clips or coils, cerebrospinal fluid shunts, bullets or pellets lodged in the head or upper body, metal plates, screws, staples or sutures in skull, neck, shoulders, arms or hands, electrodes, radioactive seeds, stents, filters, metallic heart valves, facial tattoos with metallic ink.

#### Proposed mechanism of action

The vagus nerve has several connections to higher brain centres that are important in pain regulation, such as the nucleus tractus solitarius and spinal trigeminal nucleus. Early studies suggested that the acute effect of vagus nerve stimulation is mediated by direct inhibition of afferents to the caudal trigeminal nucleus.^22^ More recently, neuroimaging studies have shown that chronic vagus nerve stimulation inhibits activation of the thalamus, limbic system, dorsal pons, locus coeruleus and nucleus tractus solitarius, all of which are structures identified in imaging studies as part of the pain matrix of headache.^23^ There is also evidence that it may inhibit pain by reducing the concentration of glutamate in the trigeminal nucleus caudalis; this in turn may reverse central sensitisation in chronic headache.^24^

### Transcranial magnetic stimulation and the SpringTMS device

Transcranial magnetic stimulation applies a brief single magnetic pulse to the scalp and underlying cortex. This pulse induces electrical fields in the cortex, altering neurotransmitter release and disrupting cortical spreading depression. The SpringTMS device is a rechargeable handheld device that delivers a single pulse of magnetic stimulation to the back of the head. As with other non-invasive devices, it is not widely available through NHS sources but individual funding requests can be made (with special arrangements for clinical governance, audit and outcome data collection, as per National Institute for Health and Care Excellence recommendations).^25^ In the private sector, it is available from the manufacturer for a free 3-month trial after which the patient must pay £158 every month for continuing use.

#### Evidence for SpringTMS

##### Acute treatment of migraine with and without aura

The evidence for acute treatment of migraine comes from only one small manufacturer-sponsored study and postmarketing surveys. A sham-controlled study on SpringTMS in the acute treatment of migraine with aura in 164 patients reported a pain-free response rate higher in those with active compared with sham treatment at both 2 h (39% vs 22%) and 24 h (29% vs 16%) for a therapeutic gain of 17% (p=0.0179).^26^ As above, in a previous trial of acute migraine treatment, the pain-free rate was 67% for 100 mg sumatriptan with a placebo rate of 28%.^20^

In an open-label postmarketing survey of 462 subjects in the UK using SpringTMS as acute treatment for migraine with and without aura, authors obtained 3-month follow-up data on only 190 patients.^27^ The discontinuation rate within the 3-month follow-up period was 55% (n=105) mainly due to inadequate benefit (n=49), cost (n=−17) or inconvenience (n=15). Of the 190 subjects who completed 3 months follow-up, 62% reported some reduction in migraine pain and 59% some reduction in attack duration. The group did not provide further clarification with responder rates and so we cannot make meaningful comparisons to other acute treatments for migraine.

##### Preventative treatment of migraine

There is no controlled evidence to support the use of SpringTMS in the prevention of migraine.

#### Possible uses of SpringTMS

From the limited evidence available, SpringTMS might have a role in the acute treatment of migraine with and without aura in those at risk of overusing acute medications or if acute pharmacological treatments are ineffective (table 1). As with the other devices, there are no clear safety data in children or in pregnancy; however, three women among the postmarketing data completed normal pregnancies.^27^ Current evidence does not support the use of SpringTMS in the preventative treatment of migraine or for the treatment of cluster headache.

#### Using SpringTMS

**Figure 4:**
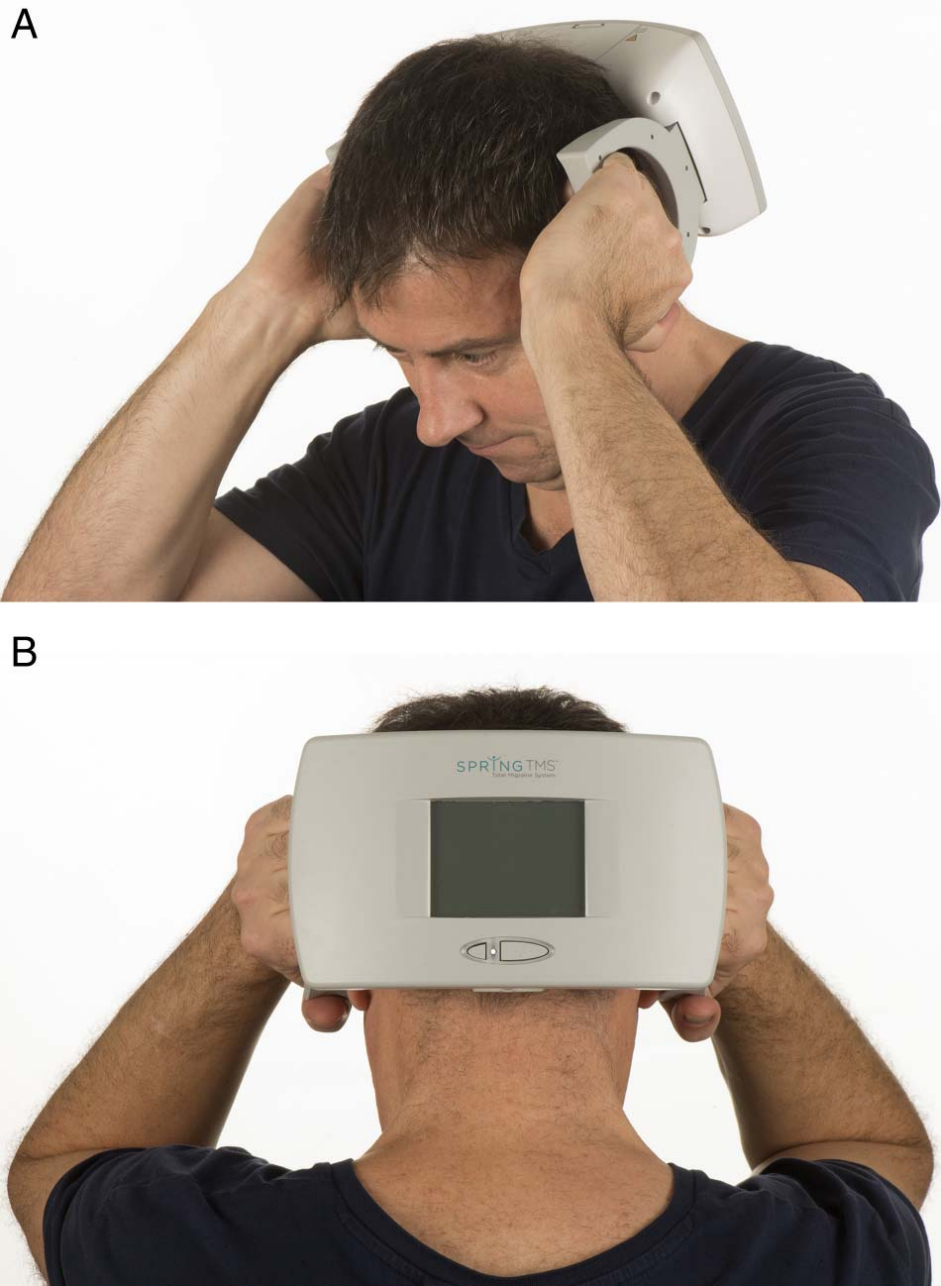
Transcranial magnetic stimulator. (A) and (B) show how a patient would use the SpringTMS device to deliver a pulse to the occipital region in order to treat acute migraine with aura attacks. Image published with permission of individual.

Box 4 gives our recommended regimens for SpringTMS in acute migraine attacks. The device is held against the occiput and the pulse delivered with the press of a button (figure 4). The device should be used as early as possible into a migraine attack. Following review, the clinician can adapt the number of pulses delivered if needed. Patients should use the device for at least 3 months to assess efficacy as there is some evidence of an increased benefit at 12 weeks compared with 6 weeks.^27^ Reported side effects are transient and mild; these include dizziness, light-headedness, tingling and worsening of migraine pain. Table 2 lists its contraindications.

Box 4 **Using SpringTMS for the acute treatment of migraine with and without aura**

Use the device at onset of migraine symptoms of pain and/or aura.

The device is held against the occiput and a button pressed to initiate the pulse.

*For acute treatment of migraine*:

Deliver two sequential pulses as early as possible into the attack.

Continue delivering two pulses every 15 min for 2 h or until attack resolves.

After first month, reassess and, if needed, increase to three pulses with each treatment.

After second month, reassess and, if needed, increase to four pulses delivered with each treatment.

#### Proposed mechanism of action

Patients with migraine are thought to have a state of brain hyperexcitability, and this has been shown in transcranial magnetic stimulation studies.^28^ This hyperexcitable cortex leads to a lowered threshold for cortical spreading depression, a wave of depolarisation of neural membranes, which has been linked to the generation of migraine aura and activation of meningeal nociceptors directly or via descending facilitation pathways.^29^ In animal studies, single-pulse transcranial magnetic stimulation inhibits cortical spreading depression. Thus, in acute migraine, single-pulse transcranial magnetic stimulation has the potential to terminate aura and reduce the severity of headache in those with aura. Repetitive transcranial magnetic stimulation reduces cortical hyperexcitability by modulating concentrations of neurotransmitters such as dopamine and glutamate in the caudate and hippocampus.^28^ Thus, repetitive transcranial magnetic stimulation may produce long-term changes in neuronal excitability, reversing central sensitisation and reducing headache frequency. However, much of the work with repetitive transcranial magnetic stimulation has been based on laboratory studies and it has not yet translated into a useful clinical treatment effect.

## Invasive neurostimulation devices

### Occipital nerve stimulation

**Figure 5:**
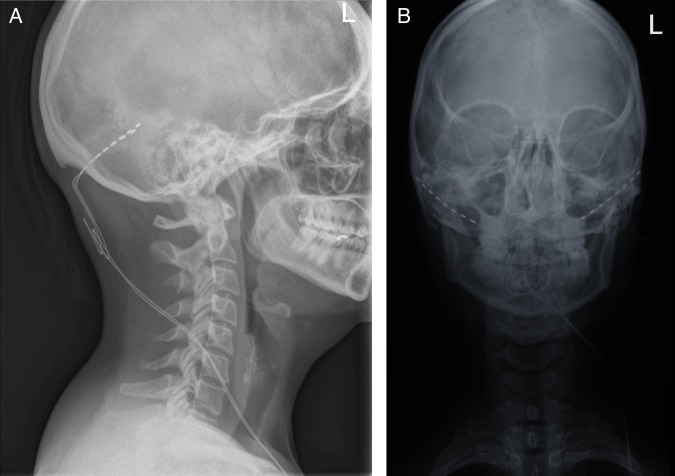
Skull X-rays showing occipital nerve stimulation electrode placement. Lateral (A) and anterior posterior (B) skull views showing the occipital nerve stimulation electrodes (dotted lines) and leads (solid lines).

The occipital nerves are a target for stimulation due to the anatomical overlap between the trigeminal and cervical afferents in the trigeminocervical complex. This allows stimulation of the occipital region to modulate pain in the trigeminal distribution. Occipital nerve stimulation is a non-destructive surgical procedure where electrodes are placed subcutaneously in the occipital region and then wired to a battery pack in the chest or abdomen (figure 5). Stimulation parameters can be adjusted to control paraesthesia. Patients can adjust their device and recharge the battery using a handheld remote control. Occipital nerve stimulation is not widely available and should only be performed in highly specialised services with established surgical and headache teams.

#### Evidence for occipital nerve stimulation

Open-label studies have shown possible efficacy in preventing chronic migraine, chronic cluster headache, hemicrania continua and short-lasting unilateral neuralgiform headache attacks.^30^ The average response rate is around 70% for chronic cluster headache and 56% for chronic migraine.

##### Preventative treatment of refractory chronic migraine

Controlled trials using occipital nerve stimulation in chronic migraine have given mixed results. The Occipital Nerve Stimulation for the Treatment of Chronic Migraine study was a randomised-controlled study of 61 subjects comparing adjustable (28 patients) and preset stimulation (16 patients) and medical management (17 patients).^31^ A positive response (a 50% reduction in monthly headache days or a >3-point reduction in pain scores) was seen in 39% of the adjustable group, 6% in the preset group and 0% in the medical group. Another randomised study of 125 subjects comparing active with sham stimulation showed no significant difference in migraine day reduction between the groups after 3 months.^32^ The third randomised trial of occipital nerve stimulation for chronic migraine looked at 157 patients. The proportion of subjects achieving a >50% reduction in daily pain scores was not significantly different in the active and sham groups (17% vs 14%). However, the reduction of headache days was significantly higher in the active group (27% vs 15%) as was the proportion of patients achieving at least a 30% reduction in pain scores (38% vs 19%). Pain specialists now widely accept that a 30% improvement in chronic pain represents a clinically meaningful improvement; thus, this study outcome favoured the use of occipital nerve stimulation.^33^ A 2015 systemic review and meta-analysis of the effectiveness of occipital nerve stimulation found that in a pooled analysis of the three above controlled trials, occipital nerve stimulation was associated with a mean reduction of 2.59 migraine days per month after 3 months compared with sham controls.^34^ However, analysis on other outcome measures was hampered by incomplete publications and poor reporting of data. Open-label follow-up data gave limited evidence of long-term efficacy.

##### Preventative treatment of chronic cluster headache

There are no controlled trials of occipital nerve stimulation in chronic cluster headache. However, positive open-label studies support its use (table 3). One recent review paper conducted a pooled analysis of the nine published studies involving 91 patients and found that patients reported an average 67% reduction of attack frequency.^30^

**Table 3:** Summary of main study outcomes for occipital nerve stimulation in chronic cluster headache

Study	Number of patients	Mean follow-up [range] (months)	Patients improved >50%	Change attack frequency (%)	Change attack severity (%)	Preventative treatment reduction*
Magis *et al*^35^ ^36^	14	36.62 [11–64]	12/14 (86%)	−94.6	+2.3	29%
Burns *et al*^37^ ^38^	14	17.5 [4–35]	10/14 (71%)	−33	+8	43% (triptans)
de Quintana-Schmidt *et al*^39^	4	6+	4/4 (100%)	−56	−48	21%
Fontaine *et al*^40^	13	14.6 [3–34]	10/13 (76%)	−68	−49	62%
Mueller 2013^56^	24	21.5 [4–47]	21/24 (88%)	−40	−38	40% reduction daily triptan dose
NHNN (authors) cohort (submitted for publication)	63	46 [2–129]	36/63 (57%)	−49	−25	83% (41% triptans)

*Figures show the percentage of patients on preventative drugs at baseline who have reduced or stopped medication at follow-up unless otherwise stated.

NHNN, National Hospital for Neurology and Neurosurgery.

#### Possible uses of occipital nerve stimulation

Occipital nerve stimulation, as with all invasive neurostimulation devices, must be reserved for those with highly medically refractory headaches who have not responded to all other treatments (table 1). The European Headache Society has guidelines on the use of invasive neurostimulation and patient selection (see online supplementary data table).^41^

10.1136/practneurol-2015-001298.supp1 Supplementary table

Occipital nerve stimulation can be considered for preventative treatment of refractory chronic migraine or cluster headaches. Patients must be assessed by a specialist team before consideration of surgery and must be deemed significantly disabled by their headaches. Following the implant, patients cannot have MRI scans and so occipital nerve stimulation should be avoided in those with coexisting conditions that may require future MRI scanning (ie, multiple sclerosis). Occipital nerve stimulation has no role in the acute treatment of migraine or cluster headache attacks.

#### Using occipital nerve stimulation

Bilateral leads should be implanted even with unilateral headaches as pain often swaps sides with unilateral stimulation. Following implantation, the device is programmed to give a comfortable level of paraesthesia in the distribution of the greater occipital nerve. The device is left on at all times, and generally patients are advised not to alter the settings unless the paraesthesia becomes painful or unnoticeable. Stimulation is not to be altered during acute attacks. Current rechargeable batteries should last around 5–7 years, and patients are advised to charge them at least once weekly to prevent system failure. It can take up to 3 months post-programming to detect any change in headache severity or frequency. It is important that patients are followed up frequently in the early postoperative period to ensure optimum stimulation settings are used. If after 1 year of continuous comfortable stimulation there has been no change, then it is highly unlikely that the patient will gain any benefit. In these patients, we switch off the device for at least 3 months before offering removal to ensure they have not failed to recognise the extent of any improvement.

Adverse event rates vary between centres but are reduced if the procedure is carried out in specialist centres.^42^ Hardware-related adverse events that may require additional surgical input include lead migration (13%), lead fracture (4%) and erosion of an electrode through the skin (4%). Other commonly reported adverse events include pain over the battery site (18%), infection (10%) and painful stimulation (17%).

#### Proposed mechanism of action

The trigeminal cervical complex is a group of brainstem and cervical spinal regions at which there is an anatomical convergence between the cervical and trigeminal systems. The trigeminal cervical complex is a key relay system for pain from the head and facial regions to higher pain processing centres in the hypothalamus, thalamus and brainstem. The exact mechanism of action of occipital nerve stimulation is unclear, and it is likely to act via a non-specific modulatory effect on pain-control systems. Fluorodeoxyglucose positron emission tomography imaging of patients with occipital nerve stimulation for chronic cluster headache found that metabolism normalised in several areas of the pain matrix following treatment.^36^ However, the ipsilateral posterior hypothalamus, which becomes activated during acute cluster headaches, continued to show hyperactivation. Similar findings with persistent hyperactivity of the dorsal rostral pons, which becomes activated in migraine, occurred with H_2_^15^O-positron emission tomography in patients undergoing occipital nerve stimulation for chronic migraine.^43^ These findings support the hypothesis that occipital nerve stimulation activates descending pain-control systems and restores equilibrium in antinociceptive pathways.

### SPG stimulation and the Pulsante SPG microstimulator

The SPG is an extracranial structure lying in the pterygopalatine fossa containing sympathetic and parasympathetic neurones. It has connections to the trigeminovascular system, superior salivatory nucleus and posterior hypothalamus—all areas that have an important role in the generation of cluster headache attacks. The Pulsante device is a miniaturised implantable neurostimulator with an integral lead and a battery. The lead is placed within the pterygopalatine fossa using a minimally invasive transoral approach. The patient controls the device using a handheld remote control. SPG stimulation is available on a research basis in two centres in the UK. The device is available in a limited capacity in some European sites, principally in Denmark and Germany.

#### Evidence base for SPG stimulation

##### Acute treatment of chronic cluster headache

A multicentre trial of 28 patients using the Pulsante device reported a significant difference in the number of resolved cluster attacks within the active compared with sham group at 15 min (67% vs 7%).^44^ After 2 months of treatment, 31% of the active group were still using acute medications compared with 77% of the sham group.

##### Preventative treatment of chronic cluster headache

Although the above study was designed to examine the acute use of SPG stimulation, 43% of subjects using the device to treat attacks reported a >50% reduction in the weekly cluster attack frequency after 2 months.

#### Possible uses of SPG stimulation

An expert consensus on the use of SPG stimulation in chronic cluster headache was published in 2014 (table 1; see online supplementary table).^45^ The procedure could be considered as an acute, and possibly a preventative, treatment for those with medically refractory chronic cluster headache who have failed all available medical treatments. At present, recommendations are that only those with strictly unilateral cluster attacks should be implanted. The device may be particularly useful in those patients who have not responded to or who have contraindications to triptans and those who have a high number of daily attacks.

#### Using SPG stimulation

**Figure 6:**
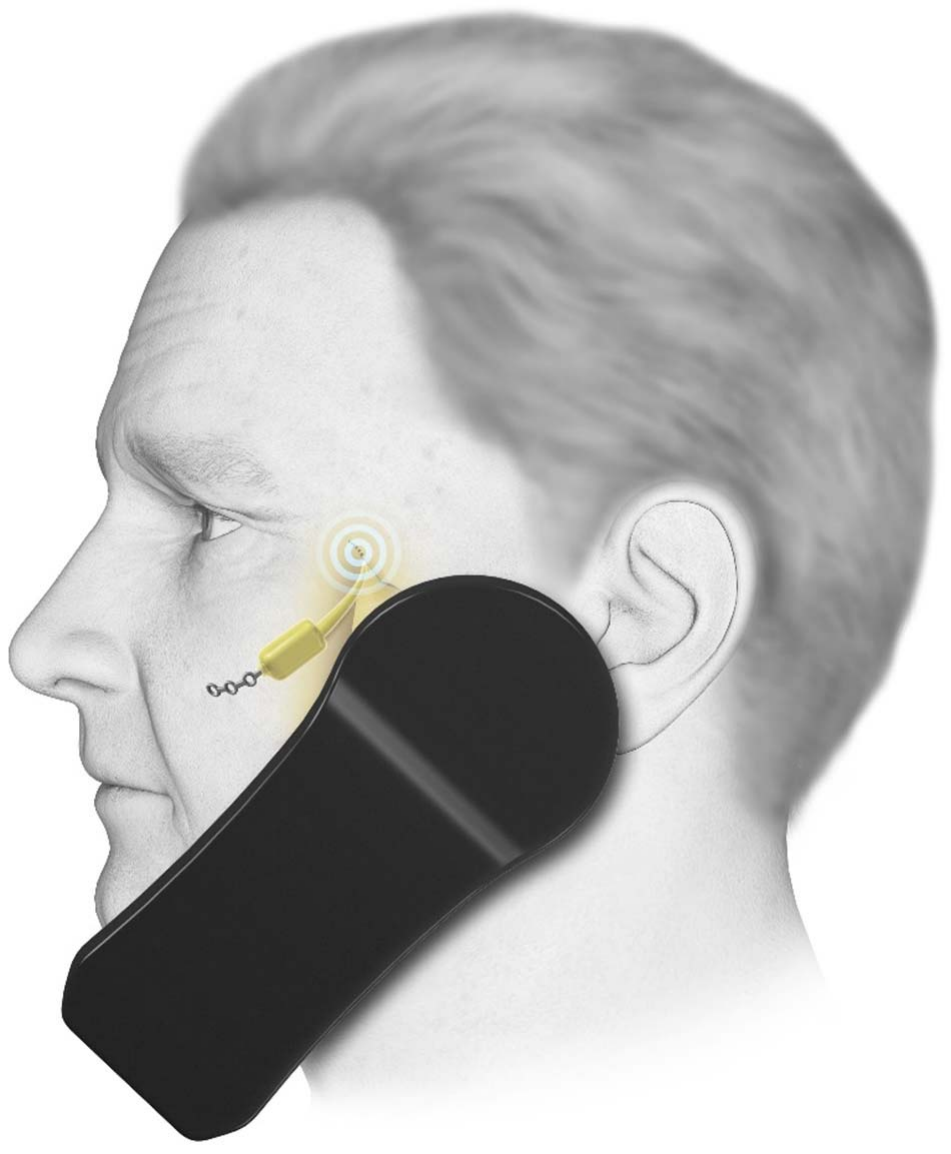
Sphenopalatine ganglion (SPG) stimulation. The SPG microstimulator (Pulsante device) in use. During an attack of cluster headache, the patient holds the handset to the cheek to activate the stimulator. Kindly reproduced with permission from ATI Technologies.

As with the other invasive neurostimulation techniques, SPG stimulation should only be carried out in specialist centres and patients must be assessed for suitability by a multidisciplinary team prior to surgery. Immediately post implant, the patients need to be reviewed on a 1–2 weekly basis to achieve stimulation settings, resulting in comfortable paraesthesia in the soft palate. At the start of an attack, the patient places the handset to the cheek above the implant and activates the device (figure 6). Stimulation should be continued for at least 15 min. After this time, if the attack continues, the patient should switch off the device and use their normal rescue medication. Even if patients stop having regular attacks, then the device could be used as a preventative giving 15 min of stimulation once or twice a day. There are ongoing studies to determine the most effective preventative regimens. Adverse events include misplacement or migration of the leads (15%), infection (6%) or mild transient sensory deficit in the maxillary division of the trigeminal nerve (81%).^44^

#### Proposed mechanism of action

Cluster headache pathology probably involves interaction between trigeminal inputs and the cranial parasympathetic outflow from the superior salivatory nucleus via the SPG. Postganglionic fibres from the SPG innervate facial structures and meningeal blood vessels. These fibres release neurotransmitters that activate trigeminal nociceptors resulting in activation of the trigeminal system, which, in turn, has a positive feedback on the parasympathetic outflow. This pathway is referred to as the trigemino-autonomic reflex.^46^ SPG stimulation probably works by interrupting this system, resulting in the termination of acute attacks via a direct effect on the trigeminal inputs and parasympathetic outflow; it may prevent attacks by inducing long-term changes in neurotransmitter release.

### Deep brain stimulation (ventral tegmentum/posterior hypothalamic region)

Functional neuroimaging techniques show that the posterior hypothalamic region is activated during cluster headache attacks.^47^ Stimulation of this region increases blood flow through areas of the pain matrix. Deep brain electrodes were first implanted in this region to treat a patient with cluster headache in 2001.^48^ Further work localised the site of implantation to the ventral tegmental area rather than the posterior hypothalamus.^49^

**Figure 7:**
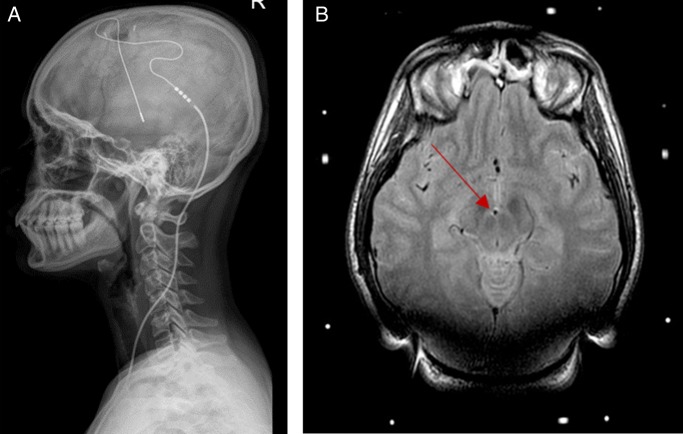
Deep brain stimulator for chronic cluster headache. (A) Skull X-ray shows placement of occipital nerve stimulation electrodes and leads. (B) Postoperative MRI brain showing occipital nerve stimulation lead in right ventral tegmental area (arrow).

Using stereotactic imaging guidance, an electrode is placed into the ventral tegmental area ipsilateral to the site of pain (figure 7). The device is programmed and kept active at all times. The method is not available for treating headache on the NHS in England.

#### Evidence for deep brain stimulation

##### Preventative treatment of chronic cluster headache

There are now >60 open-label cases of deep brain stimulation in the treatment of chronic cluster headache with an overall response rate of 66%.^30^ A recent single randomised placebo-controlled trial of 11 patients treated with deep brain stimulation in chronic cluster headache used a 1-month period of active versus sham stimulation.^50^ Although there were no differences in response rate between groups after 1 month, this may have been because of too short a follow-up period: we now know that it takes 3–6 months to see a response to deep brain stimulation.

##### Acute treatment of cluster headache

Deep brain stimulation does not help in the acute treatment of cluster attacks.^51^

#### Possible uses of deep brain stimulation

European Headache Guidelines apply to deep brain stimulation patient selection (table 1; see online supplementary data table).^41^ Patients should have proven refractory to all other treatments, including other neurostimulation techniques, and need to have normal brain anatomy. Implantation must only occur in highly specialist centres and all candidates must have been approved by a multidisciplinary team, including a psychologist. There is no evidence to support the use of deep brain stimulation in chronic migraine.

#### Using deep brain stimulation

Deep brain stimulation is reserved for patients with end-of-line refractory chronic cluster headache. Following implantation, a specialist programmes the device, which is then left switched on at all times. Unlike the other invasive neurostimulation devices, the patient is advised not to change their stimulation settings. Initially patients should be followed up 3-monthly to ensure the stimulation settings are adequate. Current implants involve a non-rechargeable battery with a lifetime of 5–7 years. As with occipital nerve stimulation, if there is no improvement after 1–2 years of stable, adequate stimulation, then the treatment is unlikely to help.

Deep brain stimulation carries the potential for serious risk. There is a single reported case of a patient with cluster headache dying from a postoperative intracerebral haemorrhage. The overall incidence of symptomatic haemorrhage in any deep brain stimulation surgery is around 2%, although our own highly experienced functional neurosurgery unit has an incidence of 0.5%.^52^ Other adverse events from our patients include transient diplopia or vertigo with changes in programming (almost universal), neck stiffness (12%) and pain over the implant (19%).

#### Proposed mechanism of action

Ventral tegmental area deep brain stimulation probably acts upon pain circuits involved maintaining chronic cluster headache.^53^ Stimulation of this region activates the hypothalamus, thalamus, somatosensory cortex, anterior cingulate, and the ipsilateral trigeminal nucleus and ganglion. These structures are also active during acute cluster attacks.^54^ On the basis that the therapeutic effect takes several weeks, it has been hypothesised that ventral tegmentum deep brain stimulation induces a functional modulation of the pain processing network in cluster headache rather than pure inhibition of hypothalamic activity.

## Conclusion

Headache syndromes are the most common disorders of the nervous system.^1^ In Europe, 1-year prevalence of adults reporting headache was 51%, migraine 14% and chronic daily headache 4%.^55^ Traditional migraine preventatives have problems with low tolerability and efficacy and so there is a demand for new treatment options. However, all the studies to date addressing neuromodulation for headache have been inadequate either due to lack of suitable placebo, small study populations, or both. Therefore, at present the conclusions that can be drawn from them are limited. Given the impact of chronic headaches on quality of life and the potential benefit that neurostimulation devices has for them, we clearly need robust randomised-controlled trials on these treatments. There are issues involved in conducting such trials, the main one being what constitutes an adequate placebo. With many of these devices, there are stimulation-related adverse effects that may unblind a trial subject—such as paraesthesia or muscle twitching—but which would be difficult to imitate with sham stimulation. Another issue with previous studies has been the use of sham stimulation where the electrical currents are below the level believed to be clinically effective. However, this level has never been studied and it is possible that studies using this method have used an active placebo, a complication in interpreting the data. From the available efficacy data, neurostimulation treatments appear similar to that of standard drugs but the major potential benefits of the treatments lie in their favourable adverse event profiles. Although current costs are high, this may be offset in some cases by reduced acute medication use (namely triptans) with successful treatment. At present, these therapies could be considered for patients with drug tolerance issues or those who have proven medically refractory. However, clinicians must discuss the limitations of these devices and the limited evidence for their use. In the future, if there is robust evidence then neurostimulation devices may take a prominent place alongside the arsenal of currently available pharmacological treatment options for headache.

**Key points**

- Controlled evidence for the use of neuro-stimulation devices is relatively sparse, particularly for the non-invasive options; however, open-label series appear to be positive, especially for invasive techniques.
- Non-invasive neurostimulation targets include the vagus nerve, supraorbital nerves and the cortex (via transcranial magnetic stimulation).
- Non-invasive devises are not routinely available on the NHS without special permissions. However their role is expanding and potentially useful.
- Invasive neurostimulation techniques included occipital nerve stimulation, sphenopalatine ganglion stimulation and ventral tegmental area deep brain stimulation.
- These techniques are reserved for those with refractory primary headache conditions only, due to cost and potential risks, and should only be conducted in highly specialist centres.
