# Supplementary material for: Neurostimulation in the treatment of primary headaches
Source: Pract Neurol. 2016 May 5;16(5):362–75. doi: 10.1136/practneurol-2015-001298 (PMC5036247; doi:10.1136/practneurol-2015-001298)
Supplement: Supplementary table [file practneurol-2015-001298supp_table.pdf]

Supplementary Table. Criteria for the use of invasive neurostimulation in primary headache [adapted from Martelletti et al [15] and Jurgens et al [21]]

Criteria for all invasive neurostimulation techniques
Patient must meet the International Headache Society criteria for chronic migraine or trigeminal autonomic cephalalgia<br>For CCH, patients should have had daily or near daily attacks for at least two years prior to stimulation<br>Patients should have been under the care of a headache specialist team for at least one year<br>All reasonable drugs must have been tried at the correct doses and for sufficient durations unless contraindicated<br>All patients should have a psychological assessment prior to surgery<br>All co-existent conditions should be identified and treated where possible prior to surgery (e.g. depression, medication overuse)<br>Patients (and doctors) must have a realistic expectation of the surgical outcome<br>Patients should be followed up by the headache specialist team for at least one year<br>Prospective headache diaries recording headache attack frequency, severity and duration as well as analgesia intake must be kept<br>Appropriate quality of life measures, disability scores and self-assessments must be kept by the patient prior and post-operatively<br>Where possible the neurostimulator should only be switched off for efficacy assessment, ideally in a double-blind fashion.<br>A clear record of adverse events is kept
Extra criteria for sphenopalatine ganglion stimulation
Attacks must occur all on one side or occurred more than 90% on one side for at least a year prior to implant<br>All candidates must undergo dental review prior to implant
